# Novel Dual Residual-Enhanced Deep Bidirectional LSTM Network for Soft Sensing of Rare Earth Component Content

**DOI:** 10.3390/s26103152

**Published:** 2026-05-16

**Authors:** Wenhao Dai, Rongxiu Lu, Pengzhan Chen, Hui Yang

**Affiliations:** 1 School of Electrical and Automation Engineering, East China Jiaotong University, Nanchang 330013, China; 8405@ecjtu.edu.cn (W.D.); 1962@ecjtu.edu.cn (H.Y.); 2 Key Laboratory of Advanced Control & Optimization of Jiangxi Province, Nanchang 330013, China; 3 School of Intelligent Manufacture, Taizhou University, Taizhou 318000, China; cyxcpz@163.com

**Keywords:** soft sensing, long short-term memory, dual residual information, rare earth component content

## Abstract

Long short-term memory (LSTM) networks demonstrate superior time-series feature extraction capabilities and have exhibited significant advantages in the soft sensing of key indicators in complex industrial processes. However, conventional LSTM networks rely solely on the output information from forward propagation through network units, neglecting the residual information between the LSTM cell outputs and the key indicators. Moreover, unidirectional LSTM networks fail to fully exploit the inherent bidirectional temporal dependencies in industrial data. These issues lead to excessive redundancy in the features learned by the network and suboptimal prediction efficiency. This paper proposes a novel dual residual-enhanced deep bidirectional LSTM (DResBiLSTM) framework that integrates bidirectional temporal modeling and dual residual learning for the soft sensing of key variables in complex industrial processes. Firstly, residual information derived from the discrepancy between previous network outputs and key indicators is introduced into the input of the traditional LSTM cell, thereby constructing a residual bidirectional LSTM (ResBiLSTM) network. Secondly, a deep neural architecture is established using residual structures to incorporate input variable residuals, enabling effective soft sensing of key industrial indicators. This framework simultaneously extracts and utilizes latent features characterized by nonlinearity and dynamics from both process and quality variables, significantly enhancing prediction performance. Finally, through both numerical simulations and experimental validations employing real-world operational data from the LaCe/PrNd solvent extraction process, the proposed method demonstrates superior predictive accuracy and better practical effectiveness compared to existing soft sensing approaches.

## 1. Introduction

In complex process industries such as rare earth extraction, the rapid detection of critical indicators (e.g., rare earth component content) plays a pivotal role in production process control and product quality assurance [1,2]. However, the inherent slow dynamics and harsh operating environments in industrial systems pose significant challenges to the direct rapid measurement of numerous core process parameters. Widely adopted offline analytical methods introduce significant delays in process optimization and quality control, thereby compromising product consistency [3,4]. Soft sensing technology leverages easily accessible secondary variables to estimate hard-to-measure quality indicators. It offers distinct advantages, such as rapid response, high prediction accuracy, and low implementation cost. Consequently, it has garnered extensive research attention and industrial adoption for quality prediction in manufacturing systems [5,6,7].

Process industries often exhibit strong nonlinearity and complex coupling relationships, making mechanism-based soft sensing models challenging to develop. In contrast, data-driven soft sensing approaches have gained significant traction with the proliferation of industrial sensors, smart instrumentation, and big data analytics [8,9,10,11]. Common data-driven methodologies encompass multivariate statistical and machine learning techniques, including partial least squares (PLS) [12], principal component regression (PCR) [13], support vector machine (SVM) [14,15], and artificial neural networks (ANNs) [16,17,18]. These approaches have been widely deployed across industrial analytics for tasks such as process monitoring, quality prediction, and fault diagnosis. However, industrial processes are inherently dynamic, and process variables frequently exhibit high dimensionality. Shallow networks often fail to capture the strong temporal dependencies in production systems, while deep architectures risk converging to suboptimal solutions due to training complexity. Compared with the shallow neural network mentioned above, deep neural networks (DNNs) are engineered to capture latent representations through cascaded nonlinear transformations, enabling the extraction of increasingly abstract latent features from raw data [19]. Deep learning architectures have achieved unprecedented performance across diverse scientific domains, including computer vision, natural language understanding, time series forecasting, and beyond [20,21]. For data-driven quality prediction, a deep residual PLS framework [22] is created to fully leverage data nonlinear information in stacked PLS residual subspaces. Deep belief networks (DBNs) [23] have demonstrated remarkable nonlinear fitting capabilities in deep learning by autonomously extracting meaningful feature representations from raw data for tasks such as classification and regression. To address data coupling challenges in industrial processes, stacked autoencoders (SAEs) [24,25] perform input data reconstruction during large-scale pretraining on industrial datasets. This enables effective feature extraction and facilitates subsequent development of soft sensing models. However, these models primarily adopt static network architectures, assuming that the observed samples are independent and identically distributed. Consequently, they struggle to effectively capture temporal dynamics information, and thus cannot adequately characterize the more pronounced nonlinearity and time-varying dynamics inherent in industrial processes.

Recurrent Neural Networks (RNNs) with time-series memory capabilities, along with their enhanced techniques, have demonstrated remarkable efficacy in processing dynamic industrial time-series data. Long short-term memory networks (LSTMs) [26,27] can effectively mitigate the gradient vanishing or exploding issues that plague traditional RNNs as the length of temporal sequences increases [28,29]. In addition to being capable of handling multi-model control and data representation, LSTMs have been widely employed in model modeling [30] and soft sensing techniques [31,32,33]. The enhanced LSTM-based variational autoencoder [34,35] demonstrates capability in extracting temporal features from complex industrial processes.

To tackle the challenge of simultaneously capturing latent features from key performance indicators (including quality information) and raw input data streams, Yuan et al. [32] proposed a supervised long short-term memory (SLSTM) network to learn quality-related latent dynamics for accurate quality prediction. In the basic SLSTM unit, the quality and input variables are simultaneously utilized to learn the dynamic hidden states, which are more relevant and useful for quality prediction. However, the real-time prediction performance of each network element in the recursive network has not been effectively utilized. Furthermore, in order to timely grasp the changes in input variables when production conditions change, Zhou et al. [36] introduced the dynamic information of input variables into the network and merged it with the nonlinear features of time series data to improve the predictive performance of the network. However, the network failed to utilize quality information to improve model performance. While these advanced LSTM variants demonstrate notable efficacy in leveraging critical variable interactions and process dynamics, they inherently suffer from a pivotal methodological gap which is the absence of residual error compensation mechanisms at the neuron level. This deficiency causes the network to retain substantial irrelevant information unrelated to key quality indicators, thereby degrading computational efficiency.

To mitigate this issue, real-time monitoring of LSTM neuron prediction performance and subsequent feedback integration into subsequent network layers becomes pivotal for optimizing prediction efficiency. Furthermore, by processing sequential data in both forward and reverse temporal contexts, bidirectional LSTM architectures demonstrate superior capability in capturing complex nonlinear temporal dependencies [37,38,39]. These structures extract multidimensional latent patterns that conventional unidirectional LSTMs may overlook, particularly in dynamic industrial environments with complex phase transitions and nonlinear system behaviors.

This paper proposes an improved BiLSTM (ResBiLSTM) network model based on network residual information, and then establishes a deep network soft sensing framework that can utilize key indicators of dual residual information of industrial process variables by introducing residual structures. The method proposed fully utilizes the dual residual architecture, which enables the model to learn both short-step dynamics and deep hierarchical features. First, by incorporating prediction residuals of key quality indicators into the LSTM framework, the proposed ResBiLSTM model feeds both input variables and neural unit output residuals into subsequent BiLSTM layers, thereby accelerating network convergence and enhancing feature extraction capabilities. Furthermore, ResBiLSTM units are further connected through residual structures to obtain a deep ResBiLSTM network. A novel dual residual-enhanced deep bidirectional LSTM network (DResBiLSTM) for soft sensor modeling is constructed for soft measurement of key indicators in complex industrial processes. To validate the proposed methodology, comprehensive experiments are conducted using both numerical datasets and real-world industrial data from LaCe/PrNd solvent extraction processes. The performance is evaluated against other soft sensing approaches. Empirical results demonstrate that the DResBiLSTM framework achieves superior prediction accuracy, particularly in scenarios involving complex multiphase extraction processes with strong nonlinear characteristics. The following are the primary contributions:

1. A novel ResBiLSTM network is proposed that can leverage the predictive performance of each LSTM unit. The discrepancy between each LSTM unit’s prediction and the ground truth is introduced into the subsequent unit’s input, enabling rapid capture of network learning effects.
2. A multi-layer ResBiLSTM network is constructed using residual architecture, where direct connections are established from the raw input to each layer’s input starting from the second layer, in addition to utilizing outputs from the preceding layer. This enhances both learning efficiency and model stability.
3. A soft sensor model for complex industrial processes is established using dual residual information, which has been applied to the soft measurement of rare earth component content in rare earth extraction processes.

This is how the rest of the article is organized. Section 2 introduces the basic LSTM and BiLSTM networks. In Section 3, the ResBiLSTM network soft sensing method based on dual residual information improvement proposed in this paper is introduced. Section 4 is allocated to validating the effectiveness of the DResBiLSTM network through numerical simulations and practical case studies on rare earth component content prediction in LaCe/PrNd extraction processes. Finally, conclusions are drawn in Section 5 based on the comprehensive discussion.

## 2. Related Works

With the significant advancements achieved by deep learning in dynamic soft sensor modeling, recurrent neural networks have been recognized as a category of network models capable of leveraging dynamic process information. These networks, which possess memory capabilities for time-series data, have been widely applied to soft sensor modeling in industrial processes. LSTM is an improved recurrent neural network that can effectively preserve the temporal features of input sequences, extract temporal dependency information of sequence data, and has temporal memory function. It also effectively solves the problem of gradient explosion or disappearance in simple recurrent neural networks, with stronger learning ability and wider applications [40,41]. The BiLSTM network consists of two layers of LSTM networks. These two layers of networks process the data in the forward and backward order of the input data sequence, respectively. Therefore, it can capture both forward and backward information in the sequence simultaneously. This section introduces LSTM and BiLSTM neural networks.

### 2.1. LSTM

LSTM is an enhanced variant of RNN that collects long short-term memory dynamic information of time series from model inputs by introducing gating mechanisms. When the model iteratively learns and propagates over time, each LSTM memory cell has a fixed weight. Therefore, it can effectively solve the problems of gradient vanishing and exploding when the network processes long time series data, and can be used to extract time series features of industrial data [42,43].

The LSTM architecture is characterized by three gating mechanisms: forget gate, input gate, and output gate. These gates are capable of maintaining both long-term and short-term memories along time series, demonstrating exceptional performance in processing time-series data from industrial processes. The LSTM cell state holds key information. Distinct logic gates regulate how this information is used. This enables long-term memory retention while also capturing short-term details. Such capabilities are essential for understanding and predicting complex extraction processes. Furthermore, nonlinear dynamics present in time-series process data are also captured by LSTM, enabling the acquisition of effective data representations through its learning mechanism.

**Figure 1:**
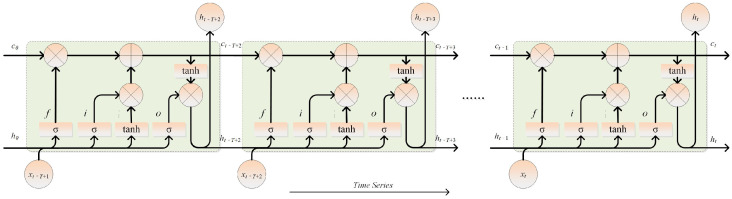
LSTM network structure.

The LSTM network structure is given in Figure 1. It is assumed that the input sequence is $X=\left\{x_{t-T+1},x_{t-T+2},\cdots ,x_{t}\right\}$, and input data from these *T* time steps are utilized to predict the current output $h_{t}$. An LSTM cell can be constructed for each time step. Taking time step *t* as an example, the state and output of its preceding LSTM cell are denoted as $c_{t-1}$ and $h_{t-1}$, respectively. The state and output of the current LSTM cell are represented as $c_{t}$ and $h_{t}$. The forward propagation of an LSTM cell is illustrated by the following equations.

(1) $$f_{t}=\sigma \left(W_{f}\left[h_{t-1},x_{t}\right]+b_{f}\right)$$

(2) $$i_{t}=\sigma \left(W_{i}\left[h_{t-1},x_{t}\right]+b_{i}\right)$$

(3) $$o_{t}=\sigma \left(W_{o}\left[h_{t-1},x_{t}\right]+b_{o}\right)$$

(4) $$\tilde{c_{t}}=\tanh\left(W_{c}\left[h_{t-1},x_{t}\right]+b_{c}\right)$$

(5) $$c_{t}=f_{t}⊙c_{t-1}+i_{t}⊙\tilde{c_{t}}$$

(6) $$h_{t}=o_{t}⊙\tanh\left(c_{t}\right)$$

where $f_{t}$, $i_{t}$, and $o_{t}$ are the forget, input, and output gates, respectively. $\tilde{c_{t}}$ is the intermediate state and $c_{t}$ is the memorized state of the LSTM cell. The network weights and biases are represented by *W* and *b*, whereas $h_{t}$ is the hidden state, or the output of the LSTM cell. σ represents the sigmoid nonlinear activation function. tanh denotes the hyperbolic tangent function, and ⊙ indicates the multiplication of the corresponding elements.

### 2.2. BiLSTM

**Figure 2:**
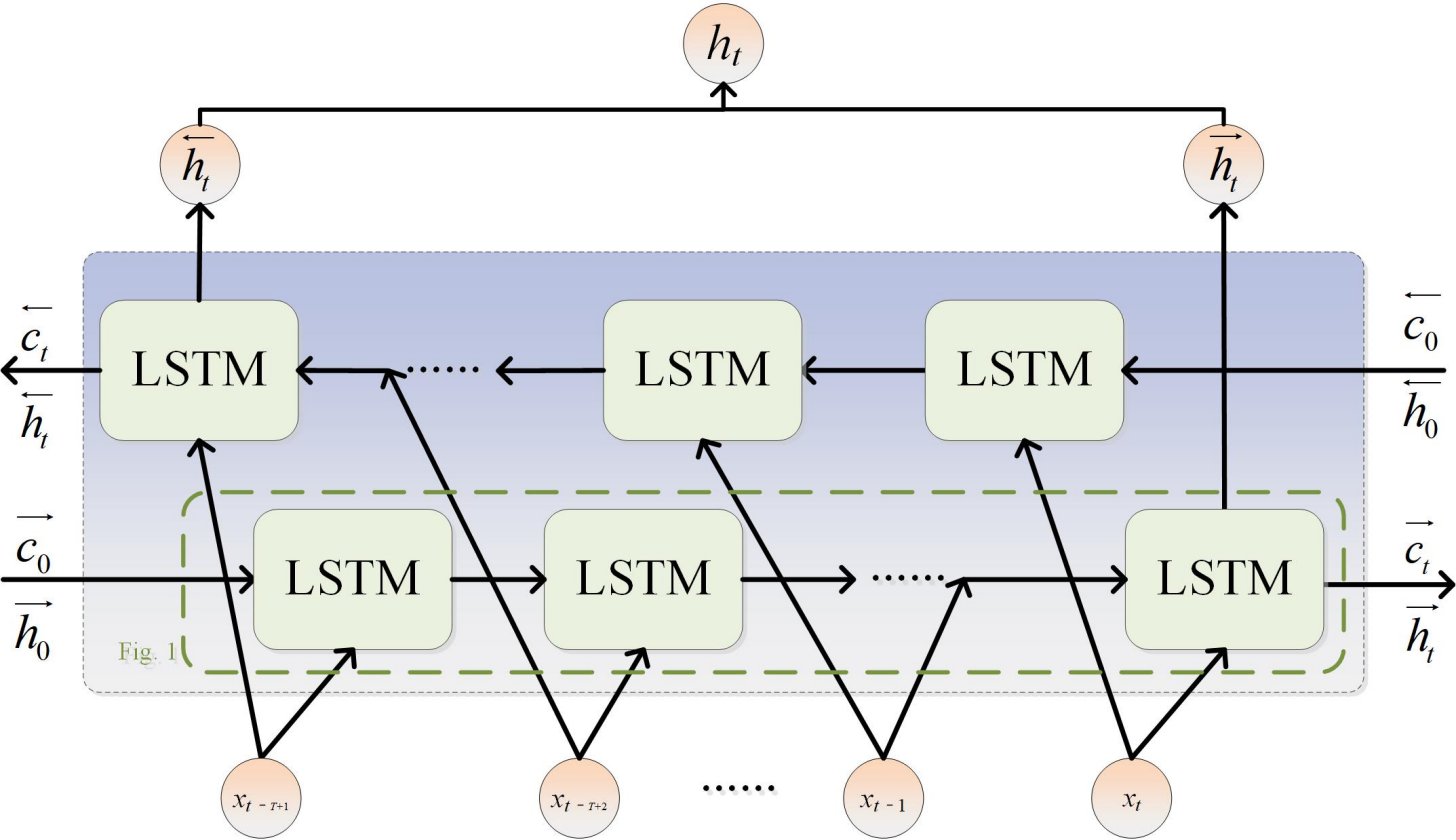
BiLSTM network structure.

The conventional LSTM architecture exclusively incorporates forward temporal information, whereas the BiLSTM network is composed of two LSTM layers. These two layers of networks process input sequences in both forward and backward directions, respectively. Therefore, it can capture both forward and backward information in the data sequence simultaneously. Owing to its unique bidirectional processing capability, the BiLSTM network demonstrates particular suitability for addressing the high-dimensional temporal dynamics inherent in complex industrial processes. The BiLSTM network structure is shown in Figure 2.

The time series’ indicated states of forward and backward propagation are represented by $\vec{h_{t}}$ and $\overset{\leftarrow }{h_{t}}$, respectively. BiLSTM obtains implicit time series features from two directions of input data and achieves higher accuracy in modeling. Potentially complex bidirectional dynamic characteristics in process variables can be discovered by utilizing the bidirectional features that are derived by Equation (7).

(7) $$h_{t}=\sigma \left(W_{h}\left[\vec{h_{t}},\overset{\leftarrow }{h_{t}}\right]+b_{h}\right)$$

where σ is the sigmoid activation function, $W_{h}$ and $b_{h}$ stand for weights and bias, respectively, and $h_{t}$ is the output value of BiLSTM.

The proposed methodology employs BiLSTM architecture that incorporates both forward and backward temporal step information, thereby enabling more effective capture of sequential data relationships. The subsequent section will introduce the proposed dual residual information-enhanced BiLSTM structure and its corresponding soft sensing methodology.

## 3. Dual Residual Information-Enhanced BiLSTM Network Soft Sensing Method

LSTM can propagate historical information through the memory cell state and hidden neuron cell state of each unit. Variants such as SLSTM [32] and DifferenceLSTM [36] further incorporate dynamic quality information and input variable variations into the LSTM architecture. However, for complex industrial processes, these approaches may not fully leverage the learning state of each unit in a timely manner. This limitation can potentially reduce network learning efficiency and prediction accuracy. Therefore, fully utilizing the learning outcome of each unit to enhance the training of subsequent LSTM units is crucial for improving the predictive performance of the model. This paper proposes an improved BiLSTM deep network soft sensing model incorporating dual residual information to enhance prediction performance. Firstly, the residual error between the predicted value and the true value from each LSTM unit is fed into the next LSTM unit. Simultaneously, a ResBiLSTM model is constructed by integrating the bidirectional LSTM structure to promptly incorporate the learning outcome of each LSTM unit into the network. Secondly, a multi-layer ResBiLSTM network is established using a residual architecture. From the second layer onwards, each layer not only utilizes the output from the preceding layer but also establishes a direct connection from the original input to the current layer’s input, thereby improving both learning efficiency and model stability. This section will introduce the ResBiLSTM unit structure and the dual residual information-enhanced deep ResBiLSTM network architecture, followed by a description of the soft sensing modeling process based on the DResBiLSTM network.

### 3.1. Improved LSTM Based on Residual Information of Key Indicators

**Figure 3:**
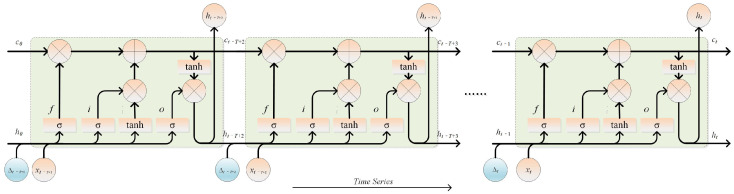
Improved LSTM unit structure based on residual information of key indicators.

Although LSTM can propagate the dynamic characteristics of industrial processes through the memory cell state and hidden neuron cell state of each unit, it cannot reveal the dynamic prediction performance of each unit and adjust network parameters in a timely manner. For complex industrial processes, due to the continuous changes in raw materials, the status of production equipment conditions, and unknown system disturbances in the system, the process parameters are constantly changing. Therefore, capturing the actual prediction performance of each network unit in real-time and incorporating prediction deviations into subsequent network units are crucial for improving network prediction efficiency and detecting key variables. This paper proposes an improved LSTM unit based on the residual information from each LSTM unit’s predictions. By introducing the residual between each network unit’s prediction and the actual value, the model’s prediction accuracy and convergence speed are enhanced. The structure of the LSTM unit improved with residual information from key indicators is illustrated in Figure 3.

As can be seen, the improved LSTM still uses LSTM unit block as basic unit. For each network cell, the residual information vector Δ is additionally used as the input of the three gates. Thus, the residual information is exploited to guide the learning of hidden and cell states. The calculation method of residual information vector Δ is as follows

(8) $$\Delta_{t-i+1}=\left|h_{t-i}-y_{t}\right|,i=1,2,\cdots ,T$$

where $h_{t-i}$ represents the output of the previous LSTM unit, $y_{t}$ denotes the true output at time *t*, and the prediction time series step is denoted by *T*. Taking time step *t* as an example, the improved LSTM forward propagation enhanced by the residual information of key indicators can be derived through the incorporation of the residual information vector Δ into Equations (1)–(4), as shown in the following equations:

(9) $$f_{t}=\sigma \left(W_{f}\left[h_{t-1},x_{t},\Delta_{t}\right]+b_{f}\right)$$

(10) $$i_{t}=\sigma \left(W_{i}\left[h_{t-1},x_{t},\Delta_{t}\right]+b_{i}\right)$$

(11) $$o_{t}=\sigma \left(W_{o}\left[h_{t-1},x_{t},\Delta_{t}\right]+b_{o}\right)$$

(12) $$\tilde{c_{t}}=\tanh\left(W_{c}\left[h_{t-1},x_{t},\Delta_{t}\right]+b_{c}\right)$$

The meanings of the symbols in these equations and the representation forms of the remaining equations are consistent with those of the standard LSTM. Similarly, by applying this method to enhance the BiLSTM network, ResBiLSTM is obtained. Through this architecture, bidirectional residual information is incorporated into both short-term and long-term states to handle complex industrial process time-series data. This structure computes residuals using true target values only at the network unit level, and these residuals are used to guide model learning within the training loss. This approach effectively improves training efficiency and helps the model converge quickly to the optimal solution. However, during model deployment, the true target is completely unavailable. In this case, the first residual calculation of the recursive network relies on true values from the historical database. For subsequent predictions, the previous step’s predicted value is used as a surrogate for the true value to compute the residual, thereby ensuring the model’s predictive effectiveness.

### 3.2. Structure of Deep ResBiLSTM Based on Residual Structure

**Figure 4:**
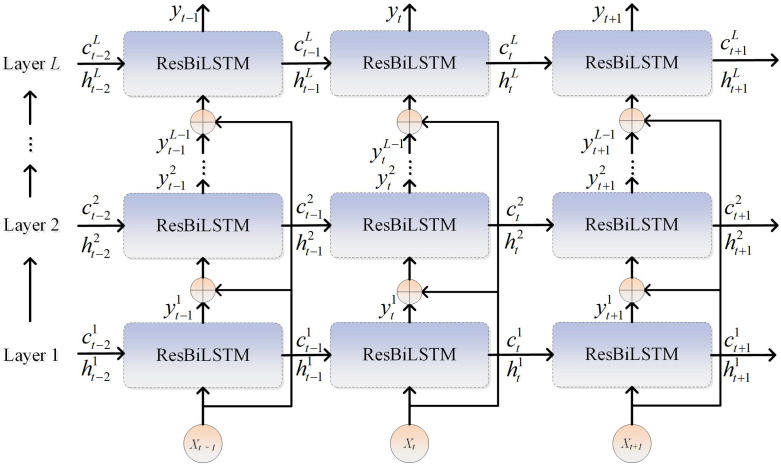
Structure of DResBiLSTM based on residual structure.

The BiLSTM units with enhanced residual information of key indicators can be utilized to construct a multi-layer ResBiLSTM network, whose architecture is illustrated in Figure 4. This forms a DResBiLSTM network capable of exploiting the dual difference information of the input variables. For each ResBiLSTM unit in the first layer, its input is the current raw data. For units in higher layers, the input of each ResBiLSTM unit includes not only the output of the previous layer but also a direct connection from the original input of the network. These two components are then fused and sent to the next layer. This architecture, which directly links the raw input to each layer of the network, enables the ResBiLSTM network to quickly learn the nonlinear high-dimensional features of the data. It enhances information flow and gradient propagation by directly feeding the original input to each layer. While the network depth increases, the original data structure remains unchanged, allowing the framework to gradually learn deep temporal information of complex industrial processes layer by layer. Subsequently, a multi-layer ResBiLSTM network is trained using backpropagation through time (BPTT).

The depth of the network is determined based on data characteristics and empirical experience. For most time-series processing models, the number of LSTM layers should not be excessively large. When the network depth exceeds three layers, gradient vanishing between layers becomes notably severe. Furthermore, due to the sequential nature of the model, the LSTM layers closer to the input suffer from slower parameter updates, resulting in significant degradation in convergence performance and efficiency, along with a heightened likelihood of falling into local minima. Conversely, an insufficient network depth fails to leverage the hierarchical data representations enabled by residual connections. To balance these trade-offs, this study employs a three-layer architecture. The number of neurons in the hidden layers is optimized via a trial-and-error process over candidate values.

### 3.3. DResBiLSTM-Based Soft Sensor

The DResBiLSTM network not only propagates the learning performance of each LSTM unit to adjust network weights in a timely manner, but also establishes residual connections from raw inputs to each layer, thereby enhancing network learning efficiency. Consequently, it can rapidly capture dynamic temporal features of industrial process data, making it highly suitable for soft sensor modeling in complex industrial processes.

**Figure 5:**
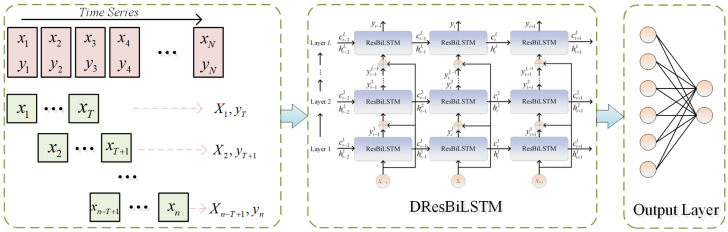
Soft sensing modeling process based on DResBiLSTM.

Figure 5 illustrates the soft sensor modeling process based on the DResBiLSTM network, which primarily involves the following modules: The first module is the network input layer. Given a dataset of industrial process data with time-series characteristics and corresponding measured values of key indicators, the input layer organizes the dataset chronologically as input to the network. The sequential input dataset is then sequentially fed into a DResBiLSTM network composed of ResBiLSTM units in chronological order. Within each ResBiLSTM unit, the nonlinear time-series dynamic features implicit in the sequential input data and corresponding reference values are continuously extracted through unit state updates and transformations via three nonlinear gates. During the network learning process, the ResBiLSTM unit dynamically adjusts network parameters based on the residuals between each unit’s output and the reference values. Subsequently, multiple ResBiLSTM layers connected through residual structures progressively learn both short-term and long-term temporal information from the time-series data, ultimately generating the network’s overall output ${\tilde{h}}_{t}$. The final part is the output layer. This layer establishes a relationship between the DResBiLSTM network’s output and the key industrial process indicator *y* through a fully connected layer, enabling the soft sensor model to predict key indicators as formulated below:

(13) $${\hat{y}}_{t}=W_{fc}{\tilde{h}}_{t}+b_{fc}$$

In this paper, the mean square error (MSE) is chosen as the loss function. The equation that follows illustrates it:

(14) $$L=MSE\left(y,\hat{y}\right)=\frac{1}{2N}\sum_{i=1}^{N}{\left(y_{i}-{\hat{y}}_{i}\right)}^{2}$$

In order to build an efficient soft sensor, gradient descent method is adopted during the training process, and Adam optimizer is used to optimize the network. The weights and bias parameters of the model are obtained by minimizing the loss function *L*. However, during model deployment, the true values of key industrial process indicators cannot be obtained in real time. Moreover, process parameters in complex industrial processes change relatively slowly. Therefore, for the established soft-sensing model, the first inference uses the average of the training set’s true values as a surrogate. In subsequent inference steps, the model’s previous prediction is used as the input for the current prediction. The soft sensor modeling algorithm based on DResBiLSTM network is shown in Table 1.

## 4. Case Study

In this section, two simulation experiments are conducted to validate the effectiveness and generalization capability of the proposed dual residual-enhanced BiLSTM network with bidirectional residual mechanisms in sequence modeling tasks. (1) A synthetic numerical example with time-series characteristics, and (2) a simulation validation of actual production data of rare earth extraction processes with complex industrial operation characteristics. The simulation procedures are executed seamlessly on a personal computer equipped with an Intel^®^ Core^TM^ i5-8250U CPU, 16 GB of RAM, and running the 64-bit Windows 10 operating system. Specifically, all models were implemented using Python 3.8.3 with PyTorch 1.11.0. Key libraries included NumPy 1.22.4 and Pandas 1.2.4. To verify model stability, all experiments were repeated using different random seeds, and the mean and standard deviation of the performance metrics were reported. The data for each model was standardized utilizing the min-max normalization technique.

Three widely used indices were computed for the testing data set in order to evaluate the prediction performance: the root mean squared error (RMSE), mean absolute error (MAE), and coefficient of determination ($R^{2}$). The following are the calculation formulas.

(15) $$RMSE=\sqrt{\frac{1}{N}\sum_{i=1}^{N}{\left(Y_{i}-Y_{i}^{\prime }\right)}^{2}}$$

(16) $$MAE=\frac{1}{N}\sum_{i=1}^{N}\left|Y_{i}-Y_{i}^{\prime }\right|$$

(17) $$R^{2}=1-\frac{\sum_{i=1}^{N}{\left(Y_{i}-Y_{i}^{\prime }\right)}^{2}}{\sum_{i=1}^{N}{\left(Y_{i}-Y_{mean}\right)}^{2}}$$

where $Y_{i}^{\prime }$ and $Y_{i}$ are the predicted and labeled quality variable values, respectively, $Y_{mean}$ is the mean of all the real values in the dataset, and *N* is the total number of test samples. The model’s prediction accuracy increases with reduced RMSE and MAE values, while the value of $R^{2}$ indicates how close the predicted value is to the true value.

### 4.1. Numerical Example

Given the proposed method’s capability to effectively process time-series data, synthetic numerical experiments incorporated fundamental functions such as sine and cosine waves that inherently exhibit temporal sequence characteristics. To account for the inevitable presence of stochastic noise in real-world industrial process data, Gaussian white noise was intentionally introduced to both input variables and system outputs during experimental design. This validation case simulated a multi-variable system with three input variables and one output variable, specifically configured to evaluate the performance of the developed soft sensing methodology. The experimental configuration adheres to the mathematical formulation presented in Equation (18), which systematically characterizes the dynamic relationships between variables.

(18) x1=tt1010+N0,0.01x2=sint+N0,0.01x3=cost+N0,0.01y=x1+51/2+x22+ex3+N0,0.01

In the mathematical formulation, the temporal variable *t* is discretized across the interval [−10, 10] with a step size of 0.05 to characterize temporal dynamics. N(0,0.01) denotes additive Gaussian white noise with zero mean and 0.01 variance. A synthetic dataset of 400 samples generated from this formulation is employed to validate the proposed methodology. The dataset is partitioned into training, validation, and testing sets with proportions of 70%, 15%, and 15% respectively. This results in 280 samples allocated for model training, 60 samples for hyperparameter tuning in the validation set, and 60 samples reserved for final performance evaluation in the testing set.

Above all, key hyperparameters need to be determined in the experimental design, and the prediction time step *T* is generally determined based on the size of the data. According to the characteristics of the data in this example, the prediction time step *T* is set to 4. The number of hidden neurons in the DResBiLSTM architecture is optimized through systematic grid search across the candidate set [10, 20, 30, 40, 50, 100, 150, 200, 250, 300], ultimately yielding an optimal configuration of 40 hidden units. The network training protocol follows the methodology outlined in Section 3.3, employing the adaptive moment estimation (Adam) optimizer with a maximum iteration limit of 300 epochs and an initial learning rate of 1 × 10^−3^. To enhance convergence stability, an adaptive learning rate decay mechanism is implemented. when validation loss exhibits no improvement over 5 consecutive training epochs, the learning rate is reduced by a factor of 0.9. Furthermore, an early stopping criterion is enforced to prevent overfitting training is terminated if validation loss plateaus for 30 successive epochs. This dual safeguard mechanism ensures robust model training while balancing convergence speed and generalization capability.

To rigorously evaluate the performance of the proposed DResBiLSTM architecture, a comprehensive benchmark analysis is conducted against state-of-the-art LSTM variants including standard LSTM, SLSTM [32], LSTM(d), and DifferenceLSTM [36]. To maintain experimental consistency across varying dataset scales, all comparative models adopt the same network architecture, following a standardized three-layer architecture consisting of input layer, hidden layer, and output layer. The hyperparameter optimization protocol maintains consistency across all comparative models. This methodological rigor ensures fair comparison while highlighting the proposed architecture’s advantages. The structure and neurons number of all models are shown in Table 2.

**Figure 6:**
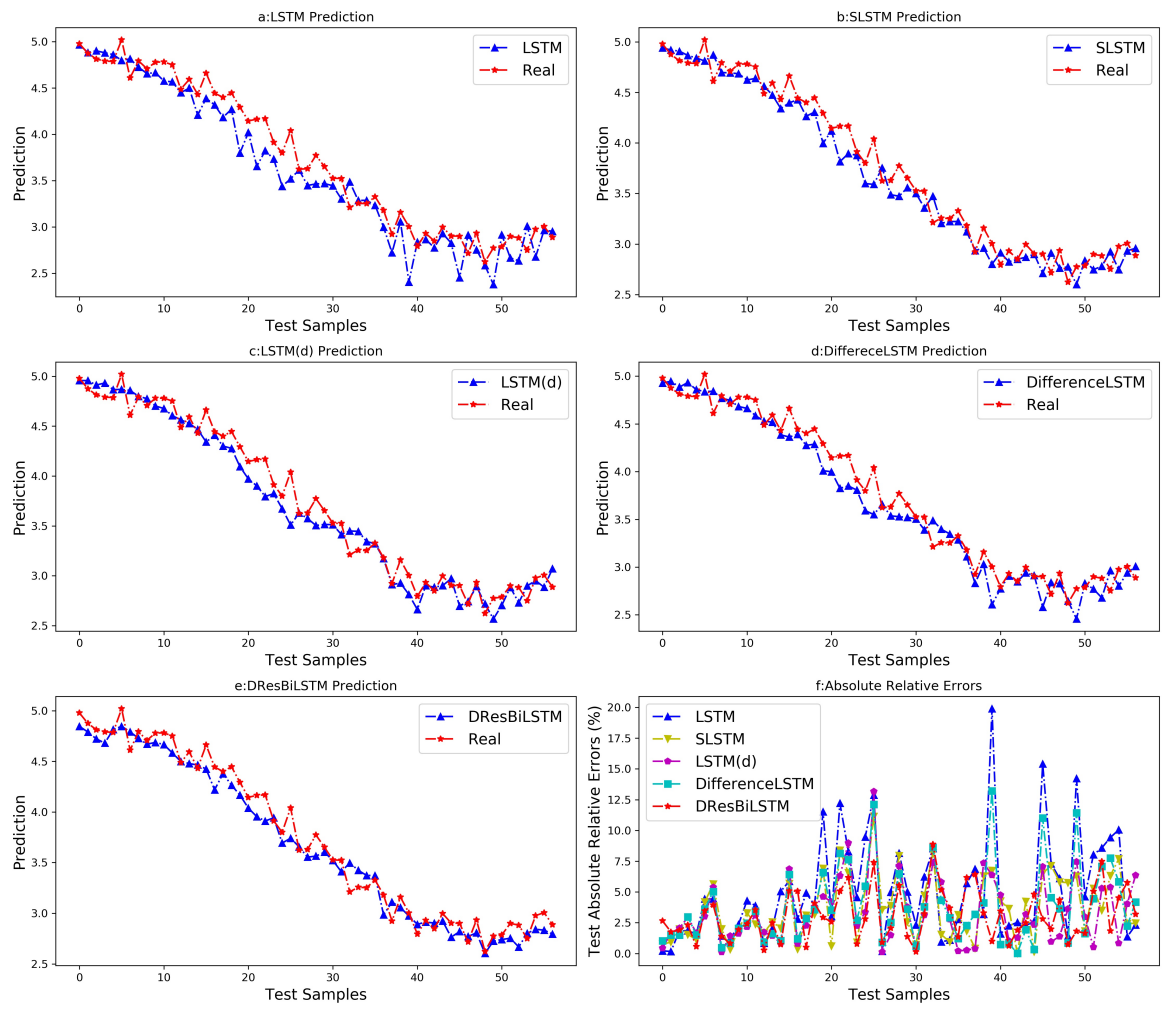
Predicted results and relative errors of all models in the numerical example.

To further analyze the predictive performance and stability of the models, ten independent experiments were conducted using different random seeds, and the average of the ten prediction results was taken as the final predicted value. The curve graphs of the average prediction results of different models are shown in Figure 6. Meanwhile, the mean values and standard deviations of ten prediction performance indicators for each model were obtained and are presented in Table 3. In the experimental results, the term before the ± symbol represents the mean, which serves as a measure of model accuracy, while the term after ± represents the standard deviation, which serves as a measure of model stability.

As illustrated in Figure 6, the proposed DResBiLSTM demonstrates remarkable consistency between predicted trajectories and ground truth values, achieving the highest prediction accuracy among all comparative models. Notably, the relative error fluctuations in the testing set exhibit superior stability compared to other architectures. Quantitative performance metrics summarized in Table 3 further validate this observation: DResBiLSTM attains the lowest RMSE (0.1492) and MAE (0.1233), while achieving the highest $R^{2}$ score (0.9634) on the independent testing set, indicating superior predictive capability and generalization performance.

First, comparative analysis of predictive accuracy among SLSTM and LSTM reveals that the supervised learning framework demonstrates superior predictive accuracy. This observation underscores the pivotal role of labeled data in enhancing model prediction precision. The SLSTM architecture achieves state-of-the-art performance by leveraging supervised data to guide the modeling processes, thereby establishing a clear advantage over its unsupervised counterparts. Similarly, the DResBiLSTM algorithm proposed in this article also uses supervised data for training and demonstrates excellent performance in prediction tasks.

Second, experimental results demonstrate that models incorporating temporal differential mechanisms, specifically DifferenceLSTM and LSTM(d), outperform conventional LSTM architectures. This performance gain validates the hypothesis that the differential information of input variables contains dynamic information embedded in the data. This residual information operator can also play a key role in modeling, which can greatly improve the predictive performance of the model.

Finally, by comparing the predictive performance of these five models, DResBiLSTM demonstrated excellent predictive performance. This advantage originates from its difference information learning paradigm, which promotes real-time propagation of prediction residual between each LSTM unit. Consequently, the timely utilization of residual information is paramount for enhancing the prediction accuracy.

In addition, to validate the effectiveness of each part in the proposed improved method, ablation experiments were conducted. The following models were compared: the traditional BiLSTM network, the cell-level residual BiLSTM (Cell-ResBiLSTM), the multi-layer residual BiLSTM (Multi-ResBiLSTM), and the proposed dual residual BiLSTM (DResBiLSTM). Using the same method as in the previous experiment, simulation verification was conducted. The prediction performance metrics of each model are shown in Table 4.

As shown in Table 4, by comparing the prediction performance metrics of each model, the cell-level improved residual BiLSTM network has smaller standard deviations in all performance metrics. This indicates that the cell-level improved network can better extract relevant hidden information from the data. As a result, it improves model stability. In contrast, using only a multi-layer residual BiLSTM network without connections from the input to each layer reduces performance. This is related to gradient issues in deep network training. On the other hand, the dual residual structure introduces residual connections into the deep network. It effectively enhances prediction performance and simulation stability.

### 4.2. Prediction of Rare Earth Component Content Based on DResBiLSTM

In order to verify the predictive performance of the method proposed in this article in actual industrial processes, it was applied to the soft sensing of rare earth element component content in rare earth extraction production processes, and the effectiveness of this model was compared with other soft sensor based models.

#### 4.2.1. Description of the Rare Earth Extraction Production Process

**Figure 7:**
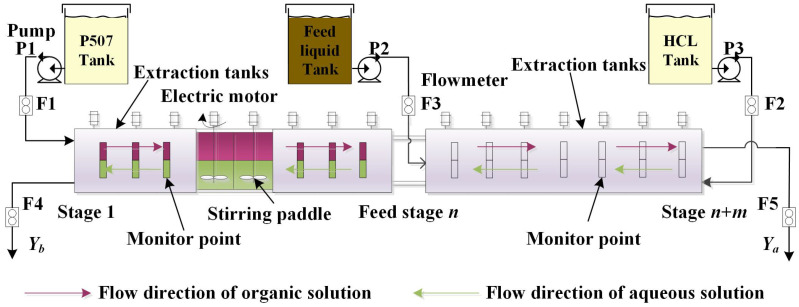
Production process description for rare earth extraction.

In the production process of rare earth extraction, efficient detection of rare earth element component content is the key to process control in the rare earth industry, which directly affects the quality of rare earth products and the improvement of production efficiency. Figure 7 is a schematic diagram of the rare earth extraction and separation production process. The rare earth material liquid is thoroughly contacted and reacted with the extraction solution P507 and detergent HCL through a cascade of continuous extraction tanks, achieving effective separation between easily extractable components and difficult to extract rare earth components.

As illustrated in Figure 7, the extractant and detergent are introduced at the initial and final stages of the extraction process, respectively, while the rare earth solution to be separated is fed at the feed stage. Under the action of stirring paddles, the organic phase solution flows from left to right, while the aqueous phase solution flows in the opposite direction, from right to left. This process is characterized by its bidirectional flow feature. Thus, the process variables implicitly contain rich bidirectional temporal characteristics. Online measurement of flow rates for all reagents and feed solution is achieved using flowmeters F1, F2, and F3. The production control of rare earth extraction primarily involves dynamic adjustment of extractant, detergent, and feed solution flow rates. To maintain stable product quality at the outlet, monitoring stages are established in each extraction and washing section. Based on the deviation between measured and target component content at these monitoring stages, the flow rates of extractant and detergent are adjusted in real-time. Consequently, the timely monitoring of component content at monitoring stages is critical for effective process control. Conventional off-line detection methods introduce significant time lags, which compromise production control efficiency. To address this challenge, the implementation of soft sensor-based prediction models for rare earth component content holds critical importance for product quality assurance. Given the constant concentrations of extractant, detergent, and feed solution under normal operating conditions, the model development selects extractant flow rate, detergent flow rate, rare earth feed flow rate, and feed composition as primary input variables. To incorporate temporal data characteristics, the immediately preceding measurements of easily extractable component and difficult to extract component at the monitoring stage are incorporated as auxiliary variables, thereby enhancing the model’s predictive accuracy. Table 5 provides detailed descriptions of these variables.

#### 4.2.2. Dataset

To verify the effectiveness of the proposed methodology, a LaCe/PrNd production line from a rare earth extraction and separation enterprise was selected as the research object. As illustrated in Figure 7, the extraction process comprises 48 stages of mixer-settler units divided into extraction and washing sections, with the feed solution introduced at stage 25. The PrNd component, classified as the easy-to-extract component, exits at stage 48, while the LaCe component, classified as the difficult-to-extract component, exits at stage 1. Flow rates of extractant $U_{1}$, Detergent $U_{2}$, and Feed liquid $U_{3}$ are continuously monitored and stored at 30-min intervals via flowmeters. The component content of easy-to-extract component $F_{a}$ and the difficult-to-extract component $F_{b}$ in the feed solution are directly obtained from feed parameters and recorded every 30 min. Monitoring stages components $Y_{a}$ and $Y_{b}$ are determined through daily offline laboratory analysis of field-sampled solutions. This results in a dataset structure where each process variable contains 48 sequential measurements corresponding to a single label value, represented by *t*, t+1, …, t+47, yielding a total of 10,560 process variable samples and 220 label samples for simulation validation. Since the raw data contain redundant information, feature extraction is required before use. Feature selection refers to selecting a subset of features based on their importance. The Permutation Importance (PIMP) method, which can evaluate the contribution of each feature to the output variable, has been widely used in industrial process soft sensing modeling [42]. After using this method for feature extraction, the input variables are mathematically represented by Equation (19).

(19) $$\begin{array}{c} X=[U_{1}\left(t\right),U_{1}\left(t+24\right),U_{1}\left(t+26\right),U_{1}\left(t+27\right),\\ U_{2}\left(t+1\right),U_{2}\left(t+2\right),U_{2}\left(t+16\right),U_{2}\left(t+34\right),\\ U_{3}\left(t+11\right),F_{a}\left(t+42\right),Y_{a}\left(t-1\right){]}^{T} \end{array}$$

Consequently, a total of 220 time-series datasets were acquired from the industrial production site. Following data normalization using the identical procedure as the numerical simulation, the preprocessed datasets were strictly partitioned into three sequential subsets: the initial 70% of samples formed the training dataset, the subsequent 15% served as the validation dataset, and the final 15% were reserved as the test dataset for performance evaluation. With regard to simulation experiment configurations, the platform architecture, neural network hyperparameter configurations, and training methodologies were maintained in full consistency with the aforementioned numerical simulation experiments.

#### 4.2.3. Results

In this case study, the method proposed is also compared with LSTM, SLSTM, LSTM(d), and DifferentialLSTM. The number of hidden neurons in each model was also optimized through systematic grid search on the same candidate set, and the final structure and number of hidden units obtained is shown in Table 6.

**Figure 8:**
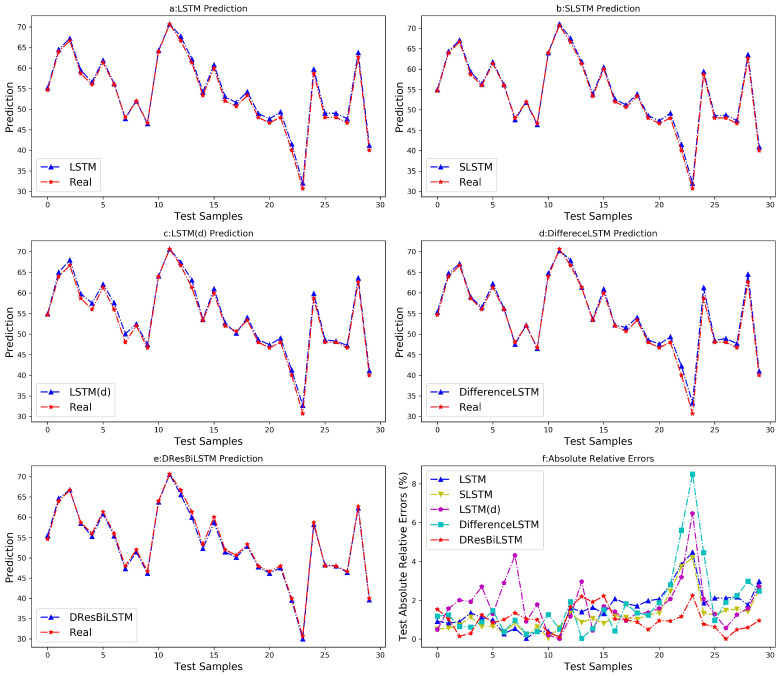
Predicted results and relative errors of all models in predicting rare earth element content.

Similarly, in order to further analyze the predictive performance and stability of the proposed model in predicting the rare earth component content, ten independent experiments were conducted using different random seeds, and the average of the ten prediction results was taken as the final prediction value. Following comprehensive model training, the predicted trajectories and relative error distributions of each model on the test set are presented in Figure 8, and the quantitative performance metrics summarized in Table 7.

As demonstrated in Figure 8, the prediction trajectory of the proposed method exhibits high consistency with the ground truth values, with remarkably stable relative error fluctuations that remain strictly within 3% across the entire test dataset. This indicates the method’s superior capability in accurately predicting rare earth component concentrations during the extraction process. All methods underwent ten repeated experiments on the test set. The mean and standard deviation of each performance metric were calculated. Table 7 further quantifies the performance disparities among algorithms. The proposed DResBiLSTM model achieves the lowest root mean squared error (RMSE = 0.6889), the lowest mean absolute error (MAE = 0.5682), and the highest coefficient of determination ($R^{2}$ = 0.9933) on the test set, outperforming conventional LSTM, SLSTM, LSTM(d), and DifferenceLSTM models. The visualization results further demonstrate that the proposed method exhibits notable advantages in trend tracking accuracy and error fluctuation control, indicating better predictive performance.

An in-depth analysis of Table 7 compares the prediction results of LSTM, SLSTM, LSTM(d), and DifferenceLSTM. It shows that LSTM(d) and DifferenceLSTM, which utilize variable difference information, exhibit poorer prediction performance than the other two models. This is due to the steady-state characteristics of the rare earth extraction process. When process variables fluctuate only slightly, the differentiation operation introduces many very small values, resulting in high-dimensional sparse input data, which actually degrades the model’s prediction performance. However, this does not mean difference information is unimportant. Notably, the proposed DResBiLSTM model strategically integrates cell-level prediction residuals into the training process through a supervised mechanism. In this way, it preserves the value of difference information while improving prediction accuracy, thus circumventing the aforementioned issue. Consequently, the proposed model not only satisfies industrial requirements but also enables timely prediction the component content of rare earth, which is crucial for maintaining stable automatic control in the rare earth extraction process.

Similarly, using the actual production data of the rare earth extraction process, ablation experiments were conducted following the same procedure as in Section 4.1. The predictive performance metrics of each model are presented in Table 8.

As shown in Table 8, comparing the prediction performance metrics of each model reveals that Multi-ResBiLSTM achieves poorer prediction results. This again demonstrates that using only a multi-layer residual BiLSTM network without input-to-each-layer connections degrades performance. This issue is related to gradient problems in deep network training. Cell-ResBiLSTM, which employs a cell-level residual structure, achieves better prediction performance than the traditional BiLSTM model. This indicates that improving the network by incorporating prediction errors at the cell level can more effectively extract relevant hidden information from the data. In contrast, the proposed dual residual structure introduces residual connections into the deep network, significantly enhancing both prediction performance and simulation stability.

In addition, to validate the effectiveness of the proposed model in predicting rare earth element component content, this study conducts comparative analyses with the traditional least squares support vector machine (LSSVM) algorithm and two enhanced approaches: (1) The mutual information (MI)-weighted LSSVM method [15] and (2) the grey relational analysis (GRA)-weighted LSSVM improvement [17]. These referenced studies achieved significant predictive performance enhancements by quantifying mutual information between data dimensions and modeling variable interrelationships, respectively. The implementation process involves two stages: First, calculating mutual information values and grey relational grades for each input variable dimension to generate distinct weighting schemes. Second, constructing corresponding weighted LSSVM prediction models based on these two strategies. The parameter configurations and optimization results of these algorithms are detailed in Table 9.

**Figure 9:**
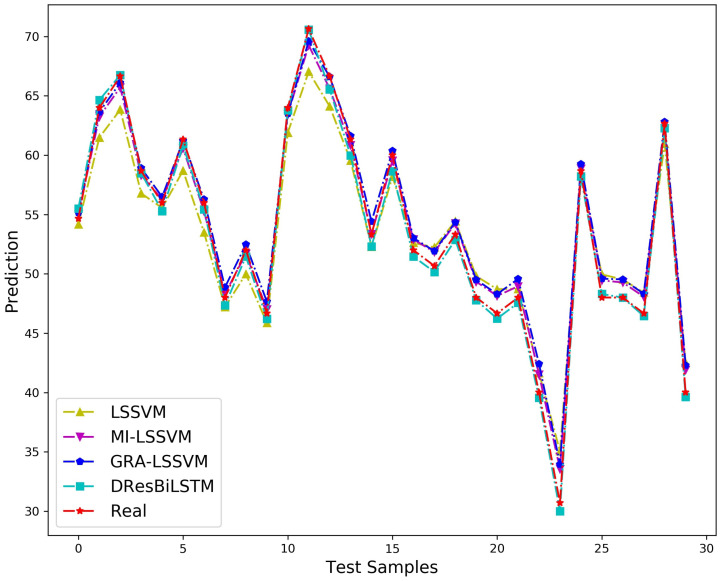
Comparison chart of prediction results of these algorithms.

The predictive performance visualization of rare earth component content models established through different methodologies on the test set is presented in Figure 9, with corresponding quantitative evaluation metrics of model performance summarized in Table 10.

As illustrated in Figure 9, the proposed DResBiLSTM model demonstrates notably superior fitting performance in predicting rare earth component content when compared to alternative methodologies. A comprehensive analysis of quantitative evaluation metrics presented in Table 10 reveals that MI-LSSVM achieves better predictive accuracy than GRA-LSSVM. This outcome suggests that the mutual information-based weighting strategy outperforms the grey relational analysis method in capturing process variable interdependencies. The performance indicators of the method employing gray relational correlation enhancement between process variables are not as good as those of the algorithm leveraging the mutual information optimization of output data. This is because the dataset comes from a stable rare earth extraction process with minimal fluctuations in process variables, where the grey relational correlation-based method shows limited effectiveness. Furthermore, the proposed DResBiLSTM network achieves substantial enhancements across all key indicators. This validates its superior ability in feature extraction and nonlinear mapping when handling temporal industrial data, as well as its capability to dynamically integrate network residual information. This makes it particularly suitable for soft sensor modeling of critical industrial process indicators.

## 5. Conclusions

In industrial processes such as rare earth extraction, each LSTM unit can predict key indicators. To fully exploit this capability, this paper introduces residual information of network prediction into the network in a timely manner. Based on this, a novel dual residual-enhanced deep bidirectional LSTM network (DResBiLSTM) is proposed for industrial process soft sensor modeling. By introducing the difference information between the predicted and true values of network units into the input of the next network unit, the network can leverage the predictive information of each network unit in a timely manner. In addition, adding direct connections from the raw input to each layer of the deep network improves learning efficiency and model stability. Then, in numerical simulations and case studies of rare earth extraction processes, the effectiveness of DResBiLSTM is demonstrated by comparing it with other related soft sensing models. Looking forward, due to the difficulty of sampling key variables in complex industrial processes, further research is needed on how to train the model of industrial process in a semi-supervised manner.

## Figures and Tables

**Table 1:** Procedure of soft sensor modeling based on DResBiLSTM.

Implementation Procedure for DResBiLSTM Based Soft Sensor
**Data preparation:** Collect measurable process and quality variables from the industrial process, extract feartures, select secondary variables, and generate datasets in chronological order. **Data preprocess:** Convert the industrial process data to the supervised sequence data with time step *T*, which is determined by prior knowledge and mechanism of industrial process, and normalize the data. **Input:** A set of supervised data $\left\{X_{1},X_{2},\cdots ,X_{N}\right\}$, $\left\{y_{1},y_{2},\cdots ,y_{N}\right\}$. **Output:** Prediction of quality variables $\left\{{\hat{y}}_{1},{\hat{y}}_{2},\cdots ,{\hat{y}}_{N}\right\}$. **Start:** **Step 1:** Split the data set into training dataset, validation dataset and test dataset by a predetermined ratio. **Step 2:** Determine the hyperparamenters of the model, including maximum number of the epochs, and initial learning rate. Determine the number of hidden neurons by using trial and error method from a candidate set. **Step 3:** Initialize the input weights and biases. **Step 4:** Update weights and biases by BPTT algorithm and Adam optimizer. **Step 5:** Repeat Step 4 under maximum epochs until convergence or obtain the early stop model. **Step 6:** Obtain the best prediction model for soft sensor application to predict the quality variables with the test dataset. **End.**

**Table 2:** The structures and numbers of neurons of all models in the numerical example.

Methods	Layers Number	Neurons Number
LSTM	3	250
SLSTM	3	20
LSTM(d)	3	300
DifferenceLSTM	3	250
DResBiLSTM	3	40

**Table 3:** Evaluation indices of all models in the numerical example.

Methods	RMSE	MAE	$R^{2}$
LSTM	0.2322 ± 2.7234 × 10^−2^	0.1841 ± 2.5687 × 10^−2^	0.9113 ± 2.0393 × 10^−2^
SLSTM	0.1678 ± 1.0450 × 10^−2^	0.1367 ± 9.1299 × 10^−3^	0.9541 ± 5.6357 × 10^−3^
LSTM(d)	0.1852 ± 6.2804 × 10^−2^	0.1485 ± 5.6328 × 10^−2^	0.9384 ± 4.9826 × 10^−2^
DifferenceLSTM	0.1795 ± 2.3560 × 10^−2^	0.1421 ± 2.1016 × 10^−2^	0.9468 ± 1.4210 × 10^−2^
DResBiLSTM	0.1492 ± 1.6217 × 10^−2^	0.1233 ± 1.5455 × 10^−2^	0.9634 ± 8.3878 × 10^−3^

**Table 4:** Evaluation indices for ablation experiments in numerical examples.

Methods	RMSE	MAE	$R^{2}$
BiLSTM	0.1672 ± 4.2361 × 10^−2^	0.1330 ± 3.4823 × 10^−2^	0.9519 ± 2.6716 × 10^−2^
Cell-ResBiLSTM	0.1714 ± 7.0372 × 10^−3^	0.1354 ± 4.7089 × 10^−3^	0.9522 ± 3.9096 × 10^−3^
Multi-ResBiLSTM	0.1984 ± 3.3276 × 10^−2^	0.1584 ± 3.0507 × 10^−2^	0.9344 ± 2.3084 × 10^−2^
DResBiLSTM	0.1492 ± 1.6217 × 10^−2^	0.1233 ± 1.5455 × 10^−2^	0.9634 ± 8.3878 × 10^−3^

**Table 5:** Variable description of rare earth extraction process.

Variable	Sampling Frequency	Description	Unit
$U_{1}$	30 min	Extractant flow rate	L/min
$U_{2}$	30 min	Detergent flow rate	L/min
$U_{3}$	30 min	Feed liquid flow rate	L/min
$F_{a}$	30 min	Content of the easy-to-extract component in the feed liquid	%
$F_{b}$	30 min	Content of the difficult-to-extract component in the feed liquid	%
$Y_{a}$	daily	Content of the easy-to-extract component in the monitoring stage	%
$Y_{b}$	daily	Content of the difficult-to-extract component in the monitoring stage	%

**Table 6:** The structures and numbers of neurons of all models in predicting rare earth component content.

Methods	Layers Number	Neurons Number
LSTM	3	300
SLSTM	3	250
LSTM(d)	3	150
DifferenceLSTM	3	200
DResBiLSTM	3	150

**Table 7:** Evaluation indices of all models in predicting rare earth element content.

Methods	RMSE	MAE	$R^{2}$
LSTM	1.1100 ± 4.7093 × 10^−1^	0.9892 ± 4.6086 × 10^−1^	0.9818 ± 1.3687 × 10^−2^
SLSTM	0.9774 ± 2.7659 × 10^−1^	0.8657 ± 2.5869 × 10^−1^	0.9870 ± 7.2514 × 10^−3^
LSTM(d)	1.4491 ± 7.9839 × 10^−1^	1.2528 ± 7.4131 × 10^−1^	0.9660 ± 3.2357 × 10^−2^
DifferenceLSTM	1.3182 ± 3.6505 × 10^−1^	1.0911 ± 3.8903 × 10^−1^	0.9764 ± 1.4065 × 10^−2^
DResBiLSTM	0.6889 ± 2.4121 × 10^−1^	0.5682 ± 2.4309 × 10^−1^	0.9933 ± 5.4795 × 10^−3^

**Table 8:** Evaluation indices for ablation experiments in predicting rare earth element content.

Methods	RMSE	MAE	$R^{2}$
BiLSTM	1.0739 ± 2.8761 × 10^−1^	0.8944 ± 2.5853 × 10^−1^	0.9844 ± 7.9284 × 10^−3^
Cell-ResBiLSTM	0.8170 ± 4.3999 × 10^−1^	0.7096 ± 4.0207 × 10^−1^	0.9893 ± 1.1361 × 10^−2^
Multi-ResBiLSTM	1.0922 ± 9.5837 × 10^−1^	0.9981 ± 9.0142 × 10^−1^	0.9743 ± 3.5177 × 10^−2^
DResBiLSTM	0.6889 ± 2.4121 × 10^−1^	0.5682 ± 2.4309 × 10^−1^	0.9933 ± 5.4795 × 10^−3^

**Table 9:** The parameter configurations of these algorithms.

Method	γ	σ	ω
LSSVM [14]	90.5484	0.0130	*
MI-LSSVM [15]	944.8938	0.5116	[0.1760, 0.1670, 0.1129, 0.1308, 0.0462, 0.0387, 0.0967, 0.0775, 0.0951, 0.0292, 0.0298]^T^
GRA-LSSVM [17]	1.5205	4.8853	[0.0926, 0.0928, 0.0925, 0.0925, 0.0884, 0.0884, 0.0982, 0.0882, 0.0782, 0.0884, 0.0999]^T^

* This variable is not available for the algorithm.

**Table 10:** Evaluation indices of these algorithms.

Methods	RMSE	MAE	$R^{2}$
LSSVM	2.0008	1.7793	0.9491
MI-LSSVM	1.0763	0.8929	0.9853
GRA-LSSVM	1.2417	0.9866	0.9804
DResBiLSTM	0.6889	0.5682	0.9933

## Data Availability

The original contributions presented in this study are included in the article. Further inquiries can be directed to the corresponding authors.

## References

[1] Lu R., Hu X., Pei C., Yang H., Dai W., Zhu J. (2025). Optimization strategy for batch-stochastic configuration network models and their application in component content prediction. Eng. Appl. Artif. Intel..

[2] Yang Z., Yao L., Shen B., Wang P. (2023). Probabilistic Fusion Model for Industrial Soft Sensing Based on Quality-Relevant Feature Clustering. IEEE Trans. Ind. Inform..

[3] Tang Y., Wang Y., Liu C., Yuan X., Wang K., Yang C. (2023). Semi-supervised LSTM with historical feature fusion attention for temporal sequence dynamic modeling in industrial processes. Eng. Appl. Artif. Intel..

[4] Dai W., Lu R., Zhu J., Chen P., Yang H. (2025). Harnessing Unlabeled Data: Enhanced Rare Earth Component Content Prediction Based on BiLSTM-Deep Autoencoder. ISA Trans..

[5] Yuan X., Li L., Shardt Y.A.W., Wang Y., Yang C. (2021). Deep Learning with Spatiotemporal Attention-Based LSTM for Industrial Soft Sensor Model Development. IEEE Trans. Ind. Electron..

[6] Yue B., Wang K., Zhu H., Yang C. (2025). A Domain-Knowledge Embedded Framework for Soft Sensing in Complex Industrial Processes with Cascading Equipment. IEEE Trans. Ind. Inform..

[7] Jia M., Zhou L., Liu Y., Gao Z., Yao Y. (2024). Global Dependency Graph Network for Soft Sensing in Process Industry. IEEE Sens. J..

[8] Lu R., Huang H., Gao G., Yang H., Liao L., Dai W. (2026). Simulation of rare earth processes based on dual multi-branch self-attentive residual deep neural networks. Measurement.

[9] Ren L., Meng Z., Wang X., Zhang L., Yang L.T. (2021). A Data-Driven Approach of Product Quality Prediction for Complex Production Systems. IEEE Trans. Ind. Inform..

[10] Ma L., Wang M., Peng K. (2023). A two-phase soft sensor modeling framework for quality prediction in industrial processes with missing data. J. Process Contr..

[11] Li L., Li N., Wang X., Zhao J., Zhang H., Jiao T. (2023). Multi-output soft sensor modeling approach for penicillin fermentation process based on features of big data. Expert Syst. Appl..

[12] Qian Q., Li M., Xu J. (2022). Dynamic prediction of multivariate functional data based on Functional Kernel Partial Least Squares. J. Process Contr..

[13] Jiao J., Zhen W., Zhu W., Wang G. (2021). Quality-Related Root Cause Diagnosis Based on Orthogonal Kernel Principal Component Regression and Transfer Entropy. IEEE Trans. Ind. Inform..

[14] Lu R., Yang H. (2015). Soft measurement for component content based on adaptive model of Pr/Nd color features. CJChE.

[15] Lu R., Rao Y., Yang H., Zhu J., Yang G. (2020). Prediction of Pr/Nd componet content based on improved just-in-time learning algorithm. Control Theory Appl..

[16] Lu R., Ye Z., Yang H., He F. (2016). Multi-RBF model prediction of Pr/Nd extraction process. CIESC J..

[17] Lu R., Deng B., Yang H., Zhu J., Yang G., Dai W. (2024). Prediction of Pr/Nd component content based on improved GRA-just-in-time learning algorithm. Control Decis..

[18] Lu R., Lai L., Yang H., Zhu J. (2023). Prediction method of CePr/Nd component content based on hybrid virtual sample. Control Decis..

[19] Zhang X., Kano M., Matsuzaki S. (2019). A comparative study of deep and shallow predictive techniques for hot metal temperature prediction in blast furnace ironmaking. Comput. Chem. Eng..

[20] Shen B., Yao L., Ge Z. (2023). Predictive Modeling with Multiresolution Pyramid VAE and Industrial Soft Sensor Applications. IEEE Trans. Cybern..

[21] Wang S., Li L., Zhang H., Liu X., Li N., Wang Q. (2024). A Local Semisupervised Soft Sensor Modeling Method Based on SAE Neural Networks for Spatiotemporal Dynamic Chemical Process. Ind. Eng. Chem. Res..

[22] Yuan X., Xu W., Wang Y., Yang C., Gui W. (2024). A Deep Residual PLS for Data-Driven Quality Prediction Modeling in Industrial Process. IEEE/CAA J. Autom. Sinica.

[23] Wang Y., Pan Z., Yuan X., Yang C., Gui W. (2020). A novel deep learning based fault diagnosis approach for chemical process with extended deep belief network. ISA Trans..

[24] Singh D., Gupta R., Kumar A., Rajendar B. (2024). Enhancing active noise control through stacked autoencoders: Training strategies, comparative analysis, and evaluation with practical setup. Eng. Appl. Artif. Intel..

[25] Zhang X., He B., Zhu H., Song Z. (2024). Information Complementary Fusion Stacked Autoencoders for Soft Sensor Applications in Multimode Industrial Processes. IEEE Trans. Ind. Inform..

[26] Yuan X., Li L., Wang K., Wang Y. (2021). Sampling-Interval-Aware LSTM for Industrial Process Soft Sensing of Dynamic Time Sequences with Irregular Sampling Measurements. IEEE Sens. J..

[27] Zheng X., Zhao Y., Peng B., Ge M., Kong Y., Zheng S. (2024). Information Filtering Unit-Based Long Short-Term Memory Network for Industrial Soft Sensor Modeling. IEEE Sens. J..

[28] Yu Y., Si X., Hu C., Zhang J. (2019). A Review of Recurrent Neural Networks: LSTM Cells and Network Architectures. Neural Comput..

[29] Al B.A., Reyes V., Olukanni T., Khalaf M., Vibho A., Pedyuk R. (2023). Advanced Misinformation Detection: A Bi-LSTM Model Optimized by Genetic Algorithms. Electronics.

[30] Huang K., Wei K., Li F., Yang C., Gui W. (2023). LSTM-MPC: A Deep Learning Based Predictive Control Method for Multimode Process Control. IEEE Trans. Ind. Electron..

[31] Lui C.F., Liu Y., Xie M. (2022). A Supervised Bidirectional Long Short-Term Memory Network for Data-Driven Dynamic Soft Sensor Modeling. IEEE Trans. Instrum. Meas..

[32] Yuan X., Li L., Wang Y. (2020). Nonlinear Dynamic Soft Sensor Modeling with Supervised Long Short-Term Memory Network. IEEE Trans. Ind. Inform..

[33] Sun C., Zhang Y., Huang G., Liu L., Hao X. (2022). A soft sensor model based on long&short-term memory dual pathways convolutional gated recurrent unit network for predicting cement specific surface area. ISA Trans..

[34] Xie W., Wang J., Xing C., Guo S., Guo M., Zhu L. (2021). Variational Autoencoder Bidirectional Long and Short-Term Memory Neural Network Soft-Sensor Model Based on Batch Training Strategy. IEEE Trans. Ind. Inform..

[35] Qin C., Wu R., Huang G., Tao J., Liu C. (2023). A novel LSTM-autoencoder and enhanced transformer-based detection method for shield machine cutterhead clogging. Sci. China Tech. Sci..

[36] Zhou J., Wang X., Yang C., Xiong W. (2022). A Novel Soft Sensor Modeling Approach Based on Difference-LSTM for Complex Industrial Process. IEEE Trans. Ind. Inform..

[37] Tornyeviadzi H.M., Mohammed H., Seidu R. (2023). Robust night flow analysis in water distribution networks: A BiLSTM deep autoencoder approach. Adv. Eng. Inform..

[38] Rathore M.S., Harsha S.P. (2022). An attention-based stacked BiLSTM framework for predicting remaining useful life of rolling bearings. Appl. Soft. Comput..

[39] Zhang M., Xu B., Jie J., Hou B., Zhou L. (2024). A Novel Bidirectional Long Short-Term Memory Network with Weighted Attention Mechanism for Industrial Soft Sensor Development. IEEE Sens. J..

[40] Ma L., Zhao Y., Wang B., Shen F. (2023). A Multistep Sequence-to-Sequence Model with Attention LSTM Neural Networks for Industrial Soft Sensor Application. IEEE Sens. J..

[41] Li Z., Li J., Wang Y., Wang K. (2019). A deep learning approach for anomaly detection based on SAE and LSTM in mechanical equipment. Int. J. Adv. Manuf. Tech..

[42] Ren L., Wang T., Laili Y., Zhang L. (2022). A Data-Driven Self-Supervised LSTM-DeepFM Model for Industrial Soft Sensor. IEEE Trans. Ind. Inform..

[43] Li Y., Peng T., Sun W., Ji C., Wang Y., Tao Z., Zhang C., Shahzad N.M. (2023). A soft sensor model based on CNN-BiLSTM and IHHO algorithm for Tennessee Eastman process. Measurement.

